# The Relative Effectiveness of Signaling Systems: Relying on External Items Reduces Signaling Accuracy while Leks Increase Accuracy

**DOI:** 10.1371/journal.pone.0091725

**Published:** 2014-03-13

**Authors:** Gavin M. Leighton

**Affiliations:** University of Miami, Department of Biology, Coral Gables, Florida, United States of America; CNRS, Université de Bourgogne, France

## Abstract

Multiple evolutionary phenomena require individual animals to assess conspecifics based on behaviors, morphology, or both. Both behavior and morphology can provide information about individuals and are often used as signals to convey information about quality, motivation, or energetic output. In certain cases, conspecific receivers of this information must rank these signaling individuals based on specific traits. The efficacy of information transfer associated within a signal is likely related to the type of trait used to signal, though few studies have investigated the relative effectiveness of contrasting signaling systems. I present a set of models that represent a large portion of signaling systems and compare them in terms of the ability of receivers to rank signalers accurately. Receivers more accurately assess signalers if the signalers use traits that do not require non-food resources; similarly, receivers more accurately ranked signalers if all the signalers could be observed simultaneously, similar to leks. Surprisingly, I also found that receivers are only slightly better at ranking signaler effort if the effort results in a cumulative structure. This series of findings suggests that receivers may attend to specific traits because the traits provide more information relative to others; and similarly, these results may explain the preponderance of morphological and behavioral display signals.

## Introduction

The social environment presents situations where alternative behavioral decisions can result in considerably different returns in inclusive fitness for the individual making the decision. To maximize fitness individuals often must observe other conspecifics and the conspecific’s set of characteristics in order to respond appropriately towards the individual being observed [1], [2]. For instance, individuals observe characteristics that indicate sex, dominance, or viability of conspecifics that then dictate behavioral decisions [3]. In many cases, individuals readily signal their condition [4] or morphological characteristics to conspecifics because it maximizes fitness [5]. Therefore, assessing conspecifics is a fundamental requirement for individual organisms and a fundamental assumption of several biological phenomena, such as direct and indirect reciprocity [6], punishment [7], and sexual selection [8], [9].

In some empirical systems ranking and remembering individuals is critical for the maintenance of sexually selected traits [10]; whether the traits are morphological features or behavioral displays, individuals are often required to sample partners and select mates based on individual characteristics [11]. While individuals that advertise their characteristics should be selected to exaggerate quality to acquire more partners, the individuals assessing potential partners should be selected to ignore uninformative traits that exploit the receiver’s sensory system [5]. The maintenance of some sexually selected traits therefore relies on these two criteria: first, individuals signal traits that convey potential direct or indirect benefits of the individual bearing the trait; and second, individuals assessing potential mates can remember and reliably rank individuals based on trait values [9]. For example, female satin bowerbirds (*Ptilonorhynchus violaceus*) visit a set of males and then re-visit a subset of original sample [12]. After sampling males, female satin bowerbirds base their mating decision on the size of the male and certain decorations in the bower [13]. As demonstrated by female bowerbirds, individuals assessing potential partners can base their behavioral decisions on some intrinsic property of a signaler such as body size [11], on some sort of external structure, such as a nest [14], [15], or both [16], [17].

In addition to sexual selection, signaling is also critical to other evolutionary mechanisms; for instance, the assessment of an individual’s behavioral output is critical to certain evolutionary mechanisms suggested to maintain cooperative behaviors [6], [18]. In direct reciprocity, an individual’s past history of behaviors must be observable so that the individuals that were previously cooperative can receive cooperation from others [19]. While definitive cases of reciprocity are uncommon, evidence suggests reciprocity could occur e.g. in chimpanzees (*Pan troglodytes*) [20], pied flycatchers (*Ficedula hypoleuca*) [21], and vampire bats [22]. A second mechanism that can maintain cooperation is punishment [7]; for punishment to maintain cooperation certain individuals must quantify behavioral output of that individual. If an individual is not cooperating, or not cooperating at a sufficient level, the individual will be attacked or evicted by others, thus rendering defection more costly than cooperation [7]. Recent concerns regarding requirements associated with tracking both the identity and behavioral output of individuals suggest punishment would be rare in nature [18]; however, punishment does seem to maintain cooperation e.g. in the cleaner wrasse (*Labroides dimidiatus*) [23].

The neural machinery necessary for identifying and observing conspecific morphology, behavior, or both [9], may be too costly for many organisms [18]. In addition to neural constraints, other characteristics of signaling systems may reduce an individual’s ability to assess conspecifics; for instance, certain traits may contain too little information to distinguish between conspecifics. A recent study on poison frogs (*Oophaga pumilio*) found that females chose to mate with the closest male, irrespective of the male’s other traits [24]. Meuche et al. [24] argued that female poison frogs chose males based on proximity because the low variance in certain male traits prevented females from effectively identifying preferable males.

While low variation may reduce the utility of a signal, several other ecological factors could potentially reduce the efficacy of image-based mechanisms (since image-based mechanisms are important in both cooperation and sexual selection, I will use the terms “signaler” and “receiver” from here on). For example, opportunities for signaling to receivers may be limited if resources are especially scarce and survival necessitates increased foraging effort. These limitations could be especially severe in species where signalers attempt to convey quality through behavior, or by acquiring objects for a display [25], [26]. In these systems, receivers are unable to assess certain morphological traits quickly; instead, individual receivers must observe signalers, and then remember the behavioral output of signalers to compare to other potential partners. In contrast, systems where signalers can build a structure that represents a cumulative effort may provide more robust and accurate estimates of an individual’s behavioral output [27], [28]. For example, the bowers of several bowerbird species [29] and the individual nests constructed by males in village weavers [30] represent the effort of males (the signaler) over time, while the time it takes females (the receiver) to assess the cumulative effort of males is a fraction of the time required for the males to build the structures.

If some traits provide more accurate information for image scoring and can be assessed in shorter time periods, receivers will be selected to use those traits as opposed to traits with low information, low variance, or large time demands [5]. These preferences can drive evolution and exploring these systems theoretically may provide insight into the evolution of certain traits. To formally investigate if specific characteristics of systems provide more accurate ranking of signalers by receivers, I designed a full factorial set of agent-based models (Table 1) that represents salient features of many signaling systems.

**Table 1:** Conceptual design of the model builds.

Signaler	Receiver	Items Needed for Display?	Example
Collection of items results in cumulative structure.	Can assess all displaying signalers in a single time step.	Yes	Village weavers^1^ (?)
Items used in display can only be observed in real time.	Can assess all displaying signalers in a single time step.	Yes	(?)
Collection of items results in cumulative structure.	Can assess one signaler in one time step.	Yes	Satin bowerbirds^2^, Wren^3^, Black Wheatear^4^, Cichlid^5^
Items used display can only be observed in real time.	Can assess one signaler in one time step.	Yes	Hangingflies^6^, other species with nuptial gifts
Total effort results in cumulative structure (morphological structures included)	Can assess all displaying signalers in a single time step.	No	Sage grouse morphological features^7^, other species with leks
Effort can only be observed in real time.	Can assess all displaying signalers in a single time step.	No	Pied flycatchers^8^
Total effort results in cumulative structure or morphology	Can assess one signaler in one time step.	No	Peacock^9^ trains, various other morphological traits
Effort can only be observed in real time.	Can assess one signaler in one time step.	No	Golden-collared manakins^10^, courtship dances, Cleaner wrasse^11^ partner monitoring

In the first column, the results of signaler effort are described and whether the displayer effort results in some structure (morphological or an external structure like a nest) that persists over time. The second column describes whether the receivers can observe the entire effort of a male in a time step or can only observe the effort in the current time step. The third column specifies whether an external item, such as a twig, needs to be found before display. In the final column putative examples of these scenarios are provided. Question marks represent examples where the author could not locate unequivocal examples of this scenario. References and scientific names printed below the table.

1 Ploceus phillipinus [30], ^2^Ptilonorhynchus violaceus [12], ^3^Troglodytes troglodytes [49], ^4^Oenanthe leucura [14],^ 5^Lamprologus callipterus, ^6^Bittacus apicalis [70], ^7^Centrocerus urophasianus [71], ^8^Ficedula hypoleuca [21], ^9^Pavo cristatus [72], ^10^Manacus vitellinus [73], ^11^Labroides dimidiatus [74].

The overall set of models tested the following questions: 1) whether constructing discrete structures (or growing specific physiological structures, as in the case of morphological features) allows for more accurate ranking of signaler output; 2) whether relying on ecologically variable items diminishes the accuracy of ranking of signaler’s output; and 3) if being able to observe all displaying signalers simultaneously (as in leks) increased accuracy of ranking of signaler output.

## Methods

### Model Design

To address these questions I built a spatially-explicit agent-based model (ABM) that had signalers perform generic display behaviors. The overall model had different builds that reflected specific situations (Table 1). In one model build the signaler’s effort did not result in a cumulative structure (as in behavioral displays), while in the second each signaler’s cumulative effort could be observed by receivers (as in discrete structures such as nests). Receivers also differed in disparate model builds; in a certain build, receivers could assess all the signaling individuals in a single time step, while in another build the receivers could only assess one “territory” at a time. Finally, in one model build signalers needed non-food display items for display, while in another build signalers could display after acquiring sufficient energy. These behavioral differences resulted in a full-factorial model design for situations that are described in Table 1.

Agent-based modeling was selected to investigate these questions because this method is amenable to modeling the set situations described above. Agent-based models allow for straightforward modeling of space [31], and consequently, agent-based models do not rely on mean-field assumptions for interactions. Specifically, agent-based models recapitulate patchiness in resources that is often a more accurate reflection of ecological conditions. Second, agent-based models allow for the development of inter-individual heterogeneity in traits [32]. Such heterogeneity is critical in these models because it allows individual receivers to potentially observe heterogeneity among signalers.

### Model Assumptions

The model assumes that receivers are observing a display trait and the display trait is left intentionally generic due to the large number of possible display traits that can be observed by receivers [33]. The model assumes a “best-of-n-males” sampling strategy [34] where receivers are observing a set of signalers, as opposed to selecting the first signaler that surpasses a certain threshold [35]. Such an assumption is warranted given the mate searching strategies seen in several taxa [12], [36], [37] and that theoretical work has demonstrated that comparing a pool of signalers can be the optimal searching strategy under certain conditions [38]. The model presented here also assumes a simple energy budget where individuals have a threshold level of energy (representing the energy needed for survival and maintenance) and the remaining energy is dedicated to display in the case of signalers or observing signalers in the case of receivers. While a more complex energy budget may be appropriate in specific situations [39], choosing any specific type of energy budget for a general model would be inappropriate as energy budgets can be highly variable between species [40]. Finally, the model assumes perfect memory, where each receiver accurately and unambiguously records the behavior of all signalers without making mistakes or forgetting information.

### Software

The agent-based model was built in Java using the compiler Eclipse © (version 1.5.1) and utilized the open-source MASON toolkit (version 16) [41]. MASON implements a premier random number generator and MASON classes allow for simple construction of spatially-explicit models. Data from model runs was output from Eclipse into text files and read into R (version 3.0.1) [42] using unique scripts that read the data and performed statistical computations.

### Statistical Assessment of Information Transfer

To quantify how accurately the receivers ranked displayers in terms of output (see Process overview and scheduling below) I needed summary statistics that averaged across receivers and simulation replicates. The specific lists output by the model were the rankings of signalers estimated by each specific receiver (20 in total) and the true rankings of signalers; to acquire the true rankings, the signalers each tracked their respective signaling effort so as to provide the true output of each signaler. Each receiver’s ranking of signalers needed to be compared to the true rankings provided by the signalers, thus suggesting the use of correlational statistics, e.g. Pearson or Spearman correlation. Due to the heterogeneity in resource acquisition by receivers, some individual receivers were never able to assess the output of displayers due to a lack of resources, resulting in no variation when receivers estimated each male’s output as 0. In these cases, traditional tests of correlation fail as they assume some sort of variation in the denominator of the test statistic. Therefore Kendall’s W, a test of concordance that can accommodate zero variance in some rankings [43], was used to assess the agreement between the true rankings of displayers and the estimates of receivers.

### Model Description

The model documentation is described according to the Overview, Design Concepts, and Details (ODD) process described in [44] and updated in [45]; the ODD method of description has been adopted because agent-based models (ABMs) have been historically difficult to describe and re-implement without the source code. The ODD contains relevant model information including variables, reproduction, and implicit assumptions. The ODD process is now utilized in multiple disciplines [45], because of its utility [46], [47]. The model code can be downloaded from the agent-based model repository OpenABM (http://www.openabm.org/model/4079/version/1/view) or from Model Code S1. A shortened ODD protocol is reproduced below that describes the model; the full ODD protocol can be found in Model ODD Protocol S1 and model validation in Model Validation S1.

### State Variables and Scales

All model runs were conducted on a 500×500 continuous space toroid; continuous space was chosen because this ABM did not rely on a lattice and the neighbor interactions that lattice designs facilitate. Time is represented using discrete time steps and advanced to time step #1440 before writing data to an external file. While slightly arbitrary, 1440 was selected because it represents the number of 15-minute time steps over the span of a month, assuming 12-hour days. In each of these time steps, the four main types of agents execute their behaviors in a random order. The four types of agents are the signalers, receivers, food items, and display items in the model builds where displayers require display items before display. Each of these agents has a specific set of variables, and there are in addition several global variables (see Table 2 for a list of model variables).

**Table 2:** List of variables and what they represent within the model.

Visibility	Variable	Description	Numeric Values
Global	Time Steps	Count of the number of time steps that have passed	Always initialized to 0 and stopped after step 1440
	Neighborhood	The spatial extent that signalers and receivers could perceive food and display items	Initialized to 10
	Energy Threshold	The lowest amount of energy a signaler or receiver could have before having to forage	Initialized to 500
Signalers	Home Location	Specific site where each signaler returned to for display	Each signaler received a unique home location
	Location	Current Location	Randomized at initialization
	Energy Reserves	Amount of energy the signaler has	Each signaler given a random value between 0 and 1000 at initialization
	Display Effort	A cumulative log of the number of time steps a signaler has displayed	Initialized to 0
	ID	A unique integer identifier for each signaler	Between 0–19 depending on the signaler
Receivers	Signaler Values	An array of values corresponding to each signaler and how much the receiver has witnessed a specific signaler display	All values in the array are initialized to 0
	Location	Current Location	Randomized at initialization
Food Items	Age	A value that increased with each time step that indicates the age of each food item.	Random values when initialized, set to 0 if born during simulation run
	Location	Current Location	Randomized at initialization
Display Items	Age	A value that increased with each time step that indicates the age of each food item.	Random values when initialized, set to 0 if born during simulation run
	Location	Current Location	Randomized at initialization

The Numeric Value column specifies how these variables were initialized, variables that were manipulated across a spectrum of values during simulations are indicated with a range of values.

### Process Overview and Scheduling

After initialization, the model is incremented in time steps in which the agents perform behaviors based on their energy reserves. In a single time step, the order of all agents is randomized to avoid order effects, and the list of randomized agents perform their specified behavior. A description of the type of agent behavior for each agent is listed below:

1. Signalers: If a signaler has sufficient energy reserves, it will return to its home territory to display. This display is dichotomous, i.e. an individual will display or it will not display regardless of the excess energy reserves. To display the signaler simply sets a Boolean display variable to “true” and the amount of energy consumed during display is the same as the amount of energy consumed in normal time steps. The model was designed this way because the model tests different display systems while simplifying other aspects of the system. If the signaler does not have enough energy, it will search for foraging resources (food items) and if there are no food items in the preset neighborhood, then it will move in a random heading to try to find food in the next time step.
2. Receivers: If a receiver has sufficient energy, it will move to a certain location to observer signalers. In the “lek” model build, the receiver moves to the center of the arena to observe all displaying signalers. In the second build, the receiver will move from one territory to the next to observe displaying signalers; and can only inspect one territory in per time step. If the receiver does not find a signaler on its territory, the receiver will move on to the next territory in the next time step. The model assumes that the receivers know where all local territories are located. To rank signalers, the receivers performed one of two disparate behaviors. In the model builds where receivers could only observe signaling in real time, the receiver would observe the signalers in the arena or territory, and for each signaling individual, increment their internal estimate for the specific signaler by 1. In the model builds where receivers could observe the entire previous effort of signalers, the receivers would obtain the true value from each signaling individual and update their internal representation of each specific signaler using the acquired value.
3. Food items and display items: Food items and display items follow the same following dynamics. Items age and if they reach the max age will die. Otherwise, the item reproduces with a 10% chance if the item “population” is under the specified carrying capacity.

### Design Concepts

#### Basic Principles

In these models I ask how fundamental features of signaling systems influence the accuracy with which receivers rank signalers.

#### Emergence

The key results expected from the model are the groups of comparisons of signaler quality produced by the several model builds. Specifically, I compared estimates of signaler quality assessed by receivers in the different signaling scenarios.

#### Adaptation

As there is no reproduction of receivers or signalers in this model, there is no adaptation.

#### Objectives

The objectives of individuals vary depending on the type of the individual. The signalers’ objective is to signal depending on the condition they are in while the objective of receivers is to assess each of the signaler’s display effort.

#### Learning

There is no learning in the model.

#### Prediction

There is no explicit prediction in the model.

#### Sensing

The signalers and receivers both sense internal levels of energy resources and respond to low resources by searching for food. While searching for food, individuals can sense food items within a predefined neighborhood. Signalers perform the same sensing procedure with display items, where they will find a display item within their neighborhood or move randomly to find an item if one is not within the search radius.

The receivers sense food using the same searching behaviors as signalers. The receivers also have to sense potential signalers that are in their neighborhood. To observe displaying signalers the receivers observe all the signalers within the arena or territory; importantly, some of the signalers in the arena or individual territory may be searching for food, therefore, the signalers must also have a Boolean display variable set to true. The Boolean variable is crucial as it only registers displaying signalers and not signalers that are within the arena foraging.

#### Interaction

There are no direct interactions among individuals. There is indirect competition for food between all the signalers and receivers. The signalers compete indirectly for the display items.

#### Stochasticity

The initialization of starting energy resources for each signaler and receiver is a random process where each individual starts with a randomly chosen integer between 0–1000 energy units. While two signaling individuals in nature may never differ in energy by three orders of magnitude, the large range is a necessary and useful component of the model. The large potential range in initial energy values should facilitate accurate rankings of signaling individuals, and in any case where the rankings are relatively inaccurate the cause of the reduced accuracy can not be attributed to reduced variation within signalers. Indeed, this randomization is important as it generates heterogeneity in both groups. There is also stochasticity in the birth of food items and display items so that ∼10% of the items will reproduce in each time step as long as these populations are under the carrying capacity.

#### Collectives

There are no collectives in this model.

#### Observation

The main data collected from the model are the true display values that all the signalers have and the values each receiver estimates for each signaler. In the output files, the array of signalers output their true values, and in subsequent columns each receiver prints their estimates for each signaler. Therefore, a 20×21 table is generated for each model run. The table represents the 20 estimates of signaler quality produced by receivers plus the true values of signaler quality. These values are then used in the assessment of concordance between signaler output and receiver rankings (see above). Finally, in model simulations where signalers require display items, a global variable was created that measured the number of time steps that a signaler had sufficient energy reserves, but could not signal because they could not locate a display item. This variable suggests whether any potential reduced accuracy in ranking is due to a lack of food or an inability to locate display items. Since this variable is only meaningful when signalers have to locate items, it was tested under the specific model builds where signalers had to locate display items before signaling.

### Initialization

At time t = 0 there are 20 signalers and 20 receivers placed randomly on the continuous space landscape. The energy reserves for each individual are the result of a random integer draw between 0 and 1000. Depending on the model conditions, between 250 and 25000 instances of each item (food and display) are created; the age for each of these items is a random integer draw from 0–100.

### Input

No external data were used as input in these models.

### Statistical Analysis of Model Simulations

The data from the simulation runs yielded 5121 data points. These data were analyzed using a general linear model in R [42]. Importantly, using parametric statistics to analyze results from agent-based model simulations is somewhat artificial as significance can almost always be achieved by increasing the number of sample runs [31]. The analysis performed here should be used to assess the effect size of each treatment (reported in Table 3), rather than using the analysis to designate each variable as significant or non-significant.

**Table 3:** General linear model coefficients for predicting Kendall’s W based on certain model characteristics.

Coefficients	Value	Standard Error	t-value	Effect size (η^2^_P_)	p-value
Intercept	0.72	0.009	83.07	na	<0.001
Food Item Production	3.02	0.126	24.02	0.250	<0.001
Items Needed for Display	−0.18	0.008	−23.13	0.261	<0.001
Signalers observed Sequentially	−0.14	0.008	−16.89	0.295	<0.001
Observe effort in real time	−0.02	0.008	−2.93	0.006	0.0034

Value represents the coefficient’s value in the predicting Kendall’s W. The effect size was estimated using partial eta_2_ (SS_Factor_/SS_Factor_+SS_Error_).

## Results

The results of the general linear model are presented in Table 3. All three factors that influenced model build significantly influenced the accuracy of receiver rankings of signalers (Table 3). Specifically, requiring signalers to acquire non-food items reduced the accuracy of display, requiring receivers to assess signalers sequentially reduced the accuracy of display, and requiring receivers to assess contemporary effort reduced the accuracy of display, though this last effect was extremely weak (Table 3).

**Figure 1:**
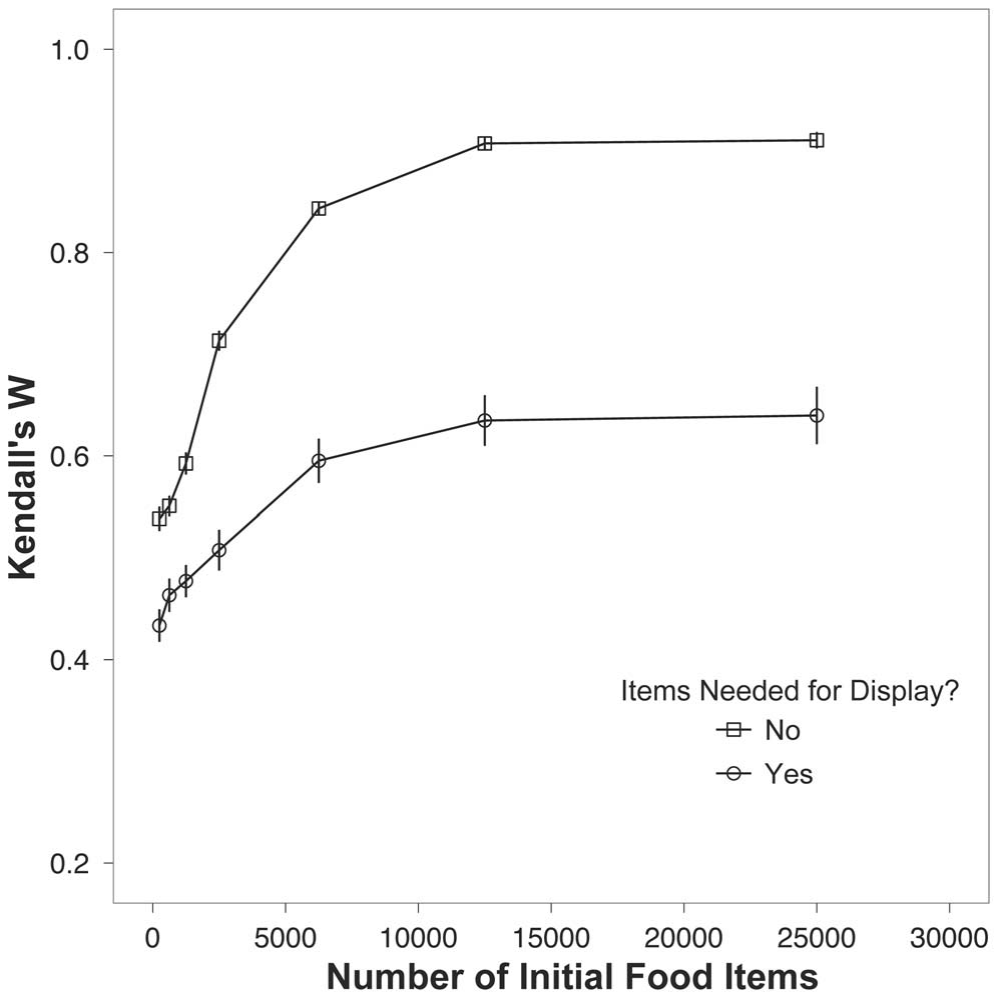
Kendall’s W for the two situations where non-food items are needed for display and not needed for display (open circles and open squares, respectively) across a range of food availability. The plotted points are the means ±1 s.e.m.

When signalers needed non-food items for display the accuracy of receiver rankings of signalers decreased considerably, i.e. the receivers were not able to assess signalers as well relative to situations where signalers did not need non-food items for display (Table 3). This pattern held at all levels of food production (Figure 1), and in both cases the accuracy of receivers’ rankings increased with increasing food production until Kendall’s W reached an asymptote.

**Figure 2:**
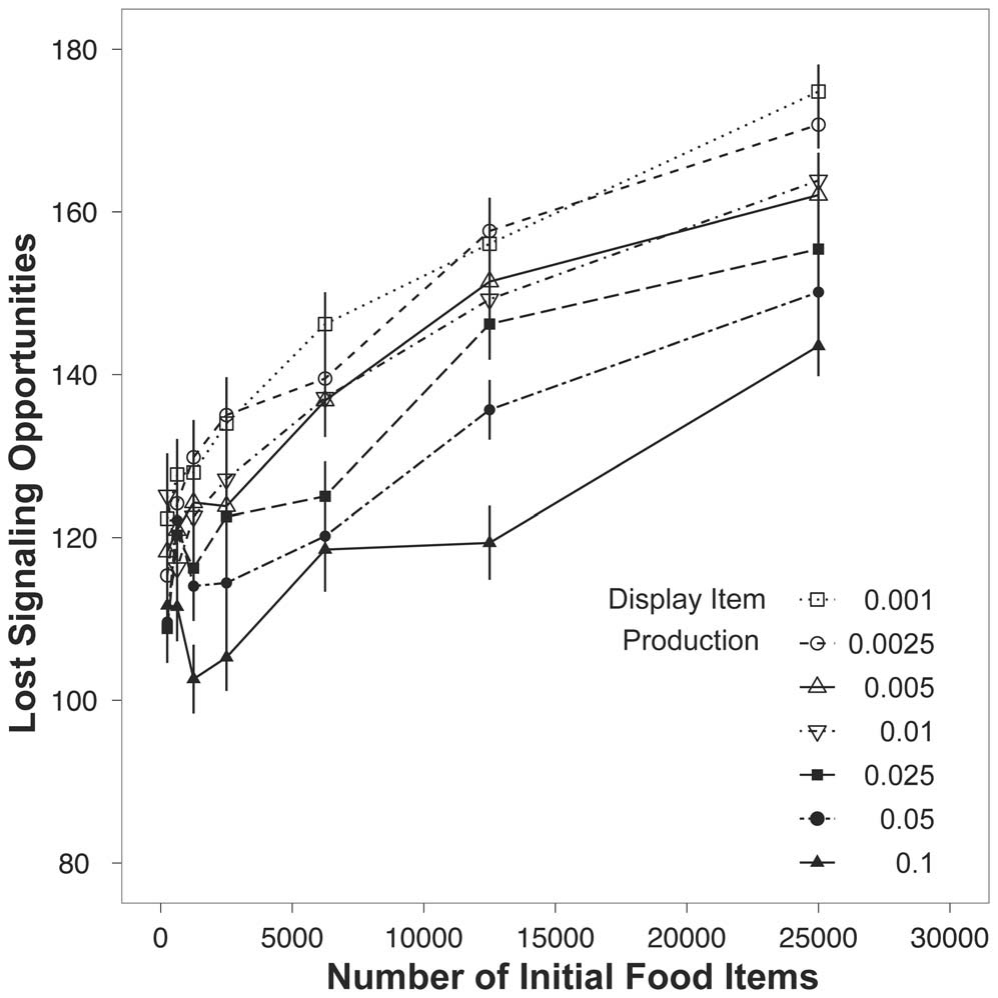
The number of lost displaying opportunities for signaling individuals in the model across food item availability and separated by the display item production. The plotted points for each display item line are the means ±1 s.e.m.

To investigate this result, a variable was created that tracked the number of time steps where signalers had sufficient energy reserves but could not signal due to an inability to locate a display item. Two factors influenced the number of lost signaling opportunities: with increasing food production, there was increased loss of opportunities for signaling; similarly, reducing the number of display items on the landscape also increased the lost opportunities for signaling (Figure 2).

**Figure 3:**
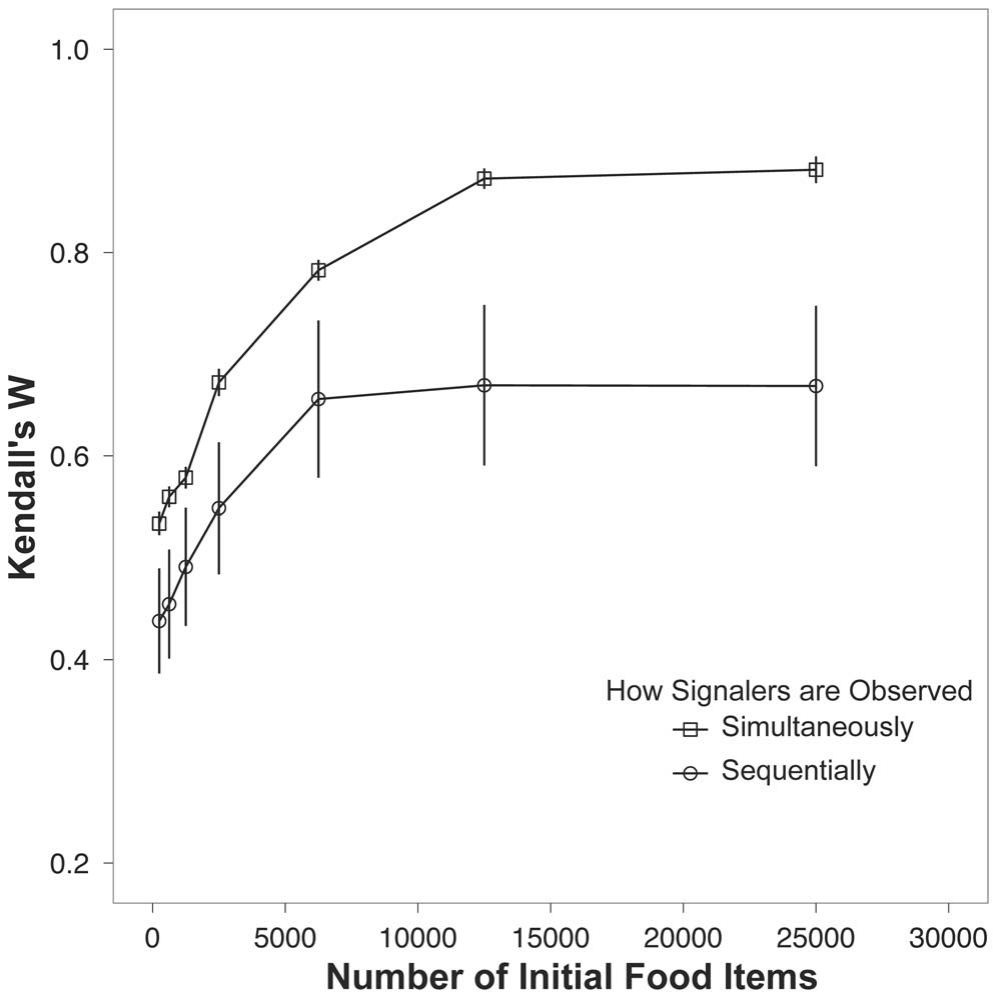
Kendall’s W for the two situations where signalers are observed either sequentially or simultaneously (open circles and open squares, respectively) across a range of food availability. The plotted points are the means ±1 s.e.m.

The second effect was the time it took receivers to sample signalers. When receivers had to assess individual signalers one at a time, the accuracy of their rankings of signalers decreased compared to scenarios where all the signalers could be observed simultaneously (Table 3). When receivers had to sample signalers sequentially, the accuracy of the receiver rankings was reduced at all resource levels (Figure 3).

**Figure 4:**
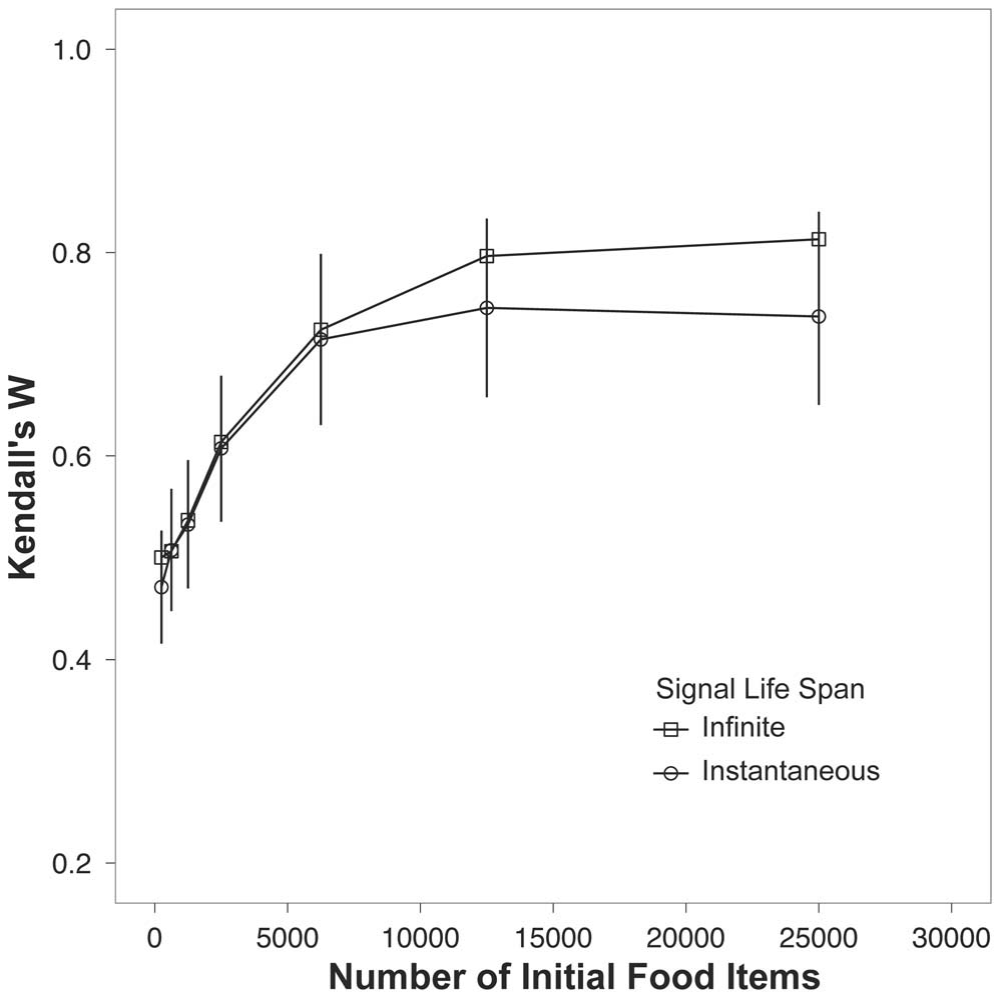
Kendall’s W for the two situations where signalers previous effort can be observed or effort can only be observed in real time (open squares and open circles, respectively) across a range of food availability. The plotted points are the means ±1 s.e.m.

The final condition compared scenarios where receivers were able to observe the entire history of a signaler’s effort, the scenario where the receiver could only observe a signaler’s display in real time. While observing the entirety of a signaler’s effort did improve estimation, the effect was weak (Table 3). Indeed, at non-saturating levels of food resources, the mean estimation of receivers is indistinguishable between the two cases (Figure 4).

**Figure 5:**
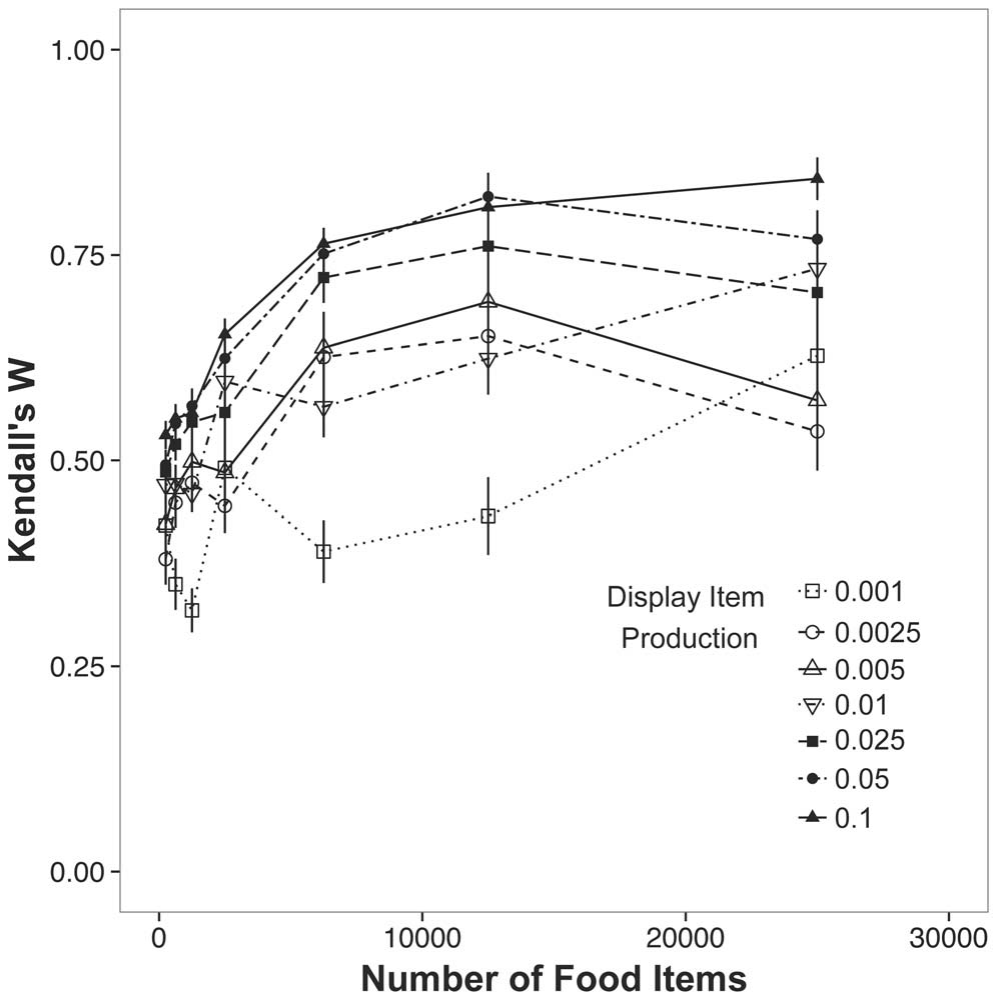
Kendall’s W for multiple display item production rates across a range of food availabilities (see legend). The plotted points are the means ±1 s.e.m.

In model runs where individuals had to acquire non-food items for display, the accuracy of assessment increased with both the number of original food items and the production of display items (Figure 5). As evidenced by the solid points, receivers were able to more accurately rank the signalers when more display items were initialized on the landscape.

## Discussion

Specific characteristics of signaling systems affect the ability of receivers to rank displaying signalers (Table 3). First, when signalers rely on external items for display the receivers do not rank displayers as accurately as when external items are not needed (Figure 1). When external items are needed for display signaling individuals lose opportunities to signal; in these situations receivers are unable to distinguish between individuals that have sufficient energy reserves to signal but can not locate a display item and those individuals that are not signaling due to inability to locate food (Figure 2). When signalers needed items for display, receivers were able to rank signalers with moderate accuracy when the landscape was saturated with both display items and food items (Figure 4), though in high food conditions the difference in accuracy between not needing and requiring display items is maximized (Figure 1). Therefore, the conditions necessary for receivers to accurately rank signalers who require non-food items for display are the same conditions that most strongly favor using signals that do not require items for display. These results question the utility of using certain behaviors as signals or cues; for instance, using nest-building behavior likely leads to lower accuracy when assessing signalers, though using the final structure can improve accuracy (see below). The scenario where receivers fail to reliably assess signalers is superficially similar to sexual selection dynamics in fallow deer (*Dama dama*). In this system, female yearlings often mate with low-ranking males and maintain more genetic variation relative to the scenario where all female fallow deer mate with high-ranking males [48].

The low accuracy of signaler ranks observed under some model conditions is also not due to memory because in all simulations the model assumes perfect memory, and no ambiguity in assigning identity to certain individuals. Given that these assumptions are almost certainly not met in nature the reliability of signaling based on acquiring items is potentially further reduced; and for these systems to function rather extraordinary mechanisms must be invoked for accurate ranking of signalers.

When receivers can assess the entire history of output of a signaler there is an increase in accuracy when ranking signalers (Figure 3). Indeed, in natural situations where non-foraging items are required for display, receivers often observe completed structures instead of observing the construction behavior that leads to the final structure. For example both bowerbirds (*Ptilonorhynchidae*) and the wren (*Troglodytes trogolodytes*) require that the display item be placed into a structure, thus extending the lifespan of the display [49].

An especially pertinent group where signalers may require external items is the weaver clade (*Ploceidae*). Many species of weaver construct elaborate nests that are used to attract mates, raise young, or both. In village weavers (*Ploceus cucullatus*) females are attracted to nests and inspect the nests of males, and manipulated nests lead to lower mating success [30], though this is confounded with lower display effort by males [50]. Across the *Ploceidae* group though, sexual selection for nest building is variable and ambiguous. In red bishops (*Euplectes orix*), evidence suggests that nest building behavior may be a signal [51]; but in baya weavers (*Ploceus philippinus*), females are more attentive to the location of nests, as opposed to the nest itself [52], and do not seem to consider the building behavior. In situations where individuals do use cumulative structures as signals, competition among signalers may select for destructive behaviors. Specifically, signalers may be expected to destroy the signaling structures built by rivals. Indeed, evidence from satin bowerbirds and village weavers suggest that males will destroy the structures of others [53], effectively erasing the record of previous effort.

When receivers can assess all of the displaying signalers at once, the accuracy of assessment is higher than when receivers had to assess signalers sequentially (Figure 2). Indeed, in species where signalers display in leks [54] females can observe a group of males in a short amount of time compared to situations where females search out males that are defending territories that are relatively far apart [55]. Interestingly, the improved accuracy of ranking males may provide an alternative explanation for the evolution of some leks. Assuming that females trade off the costs searching for mates against the benefits of choosing high quality mates [34], [56], females that select males from within aggregations may survive at higher rates than females that do not prefer aggregations. If females more accurately rank males in leks, and the most successful males acquire the majority of mating in leks [54], then the genes for attractive male displays may fall into linkage disequilibrium with genes for displaying in congregations; thus satisfying the requirements for run-away selection [57]. A female preference for males that form groups before display is evolutionarily plausible. Selection could utilize pre-existing genetic architecture for pro-social tendencies [58] in a new context [59]; specifically, a female preference for joining a group of individuals would be linked with other mating behaviors.

In this set of models signalers only displayed a singular, non-descript trait, despite the considerable evidence that many organisms utilize multi-modal signaling to communicate quality [60], [61]. If individuals employ multiple signals for redundancy [62] then the results presented here are still largely applicable. In cases where individuals use multiple signals to convey different information [63] selection to communicate non-redundant information may lead to the evolution of traits that are sub-optimal in terms of information transfer.

The results presented here provide a potential explanation for the preponderance of morphological and stereotyped displays used in sexual selection [9]. First, neither morphological structures nor stereotyped behavioral displays tend to require non-food items, allowing for increased accuracy in ranking signalers. Additionally, multiple types of these kinds of traits can be assessed in short time periods. Previous theoretical work on signaling specifies that multiple displays can evolve so long as the secondary preferences that evolve do not incur synergistic costs [64], [65]. Assessing multiple morphological traits likely requires little extra cost relative to devoting considerable portions of time to observe signaling behaviors that reduce time for maintenance behaviors such as foraging and preening. In contrast, assessing behavioral traits that rely on external items could drastically increase the search cost and thus make behavioral signals that rely on external items evolutionarily untenable [66]. However, if receivers use traits that are not useful for ranking signalers only as threshold traits, i.e. they only assess signalers that have acquired items or are present on a territory, this may reduce search costs and facilitate the evolution of multi-component signaling [65]. For instance, European bitterlings (*Rhodeus sericeus*) females choose to initially inspect males based on behavior and morphology, but final mating decisions are based on aspects of the male’s territory [67].

In cases where individuals do build structures, observing the final product is likely more efficient because the structure represents a record of output. The distinction between observing the final structure and observing the behavior that produced the structure is important. Some nests may take hours or days worth of work to build and receivers likely could not observe the entire behavioral output of multiple males. By observing the completed structure, receivers can spend considerably less time at each signaler and therefore sample more potential partners while still observing a structure that represents considerable output. Similarly, receivers can more accurately assess signalers when groups of signalers can be assessed in single time steps; as is possible in lekking species. By selecting partners from groups of signaling individuals, receivers are able to increase the number of potential partners they sample. Since many signaling systems rely on the relatively efficient characteristics identified above these results suggest that ecological and social characteristics drive selection in receivers to identify and use signals that reflect the quality of signalers [5], [68].

For selection to maintain any trait in a population the selection coefficient has to be sufficiently large given a specific population size [59]. In many animals, small population size requires a relatively large selection coefficient to maintain traits via selection [69]. Therefore, those traits that are unreliable in terms of ranking individuals will be harder to maintain via selection or will be subject to drift. The models described here have shown that certain traits are unlikely to be useful for ranking signalers, especially those traits that require external items for display. For traits that do require external items, future research should examine whether these traits are used as to make a binary choice between assessing or not assessing other aspects of a signaler. In contrast, leks are conducive to ranking males and the benefit provided by leks may provide an explanation for the evolution of leks in certain species.

## Supporting Information

Model Code S1 **The Java code necessary to compile the model in Eclipse.** To compile the model in Eclipse the package name would have to be adjusted on the local machine to match the class references.
(ZIP)

Model ODD Protocol S1 **The Overview, Design concepts, and Details (ODD) protocol for the model published here.** This is the full ODD protocol and contains extra details relative to the abridged ODD protocol in the methods.
(DOCX)

Model Validation S1 **The model validation describes the checks that were run on the model.** The main components of the model were rigorously tested to confirm that the model was functioning as described.
(DOCX)
