# Supplementary material for: The Relative Effectiveness of Signaling Systems: Relying on External Items Reduces Signaling Accuracy while Leks Increase Accuracy
Source: PLoS One. 2014 Mar 13;9(3):e91725. doi: 10.1371/journal.pone.0091725 (PMC3953539; doi:10.1371/journal.pone.0091725)
Supplement: Model ODD Protocol S1 — The Overview, Design concepts, and Details (ODD) protocol for the model published here. This is the full ODD protocol and contains extra details relative to the abridged ODD protocol in the methods. (DOCX) [file pone.0091725.s002.docx]

ODD for Display Assessment ABMs

Discrete Items Enhance Reputation Assessment

Gavin M. Leighton^1^

^1^University of Miami, Department of Biology, 1301 Memorial Drive, Coral Gales, FL

Corresponding e-mail: [gleighton@bio.miami.edu](mailto:gleighton@bio.miami.edu)

**Overview**

*1. Purpose*

Both sexual selection and cooperation are phenomena that have commanded immense attention and research in biology. In sexual selection, and in many cooperative mechanisms, behaviors of individuals are dictated by the reputation, morphological features, or traits of potential receivers of behavior. Mechanisms that maintain cooperation, such as punishment, direct, and indirect reciprocity rely on the assessment of individual reputation. Similarly, many systems that display sexual selection rely on the assessment of other individuals either for intrasexual competition or for inter-sexual choice.

However, little theoretical work has scrutinized the types of systems that most readily lend themselves to accurate assessment of conspecifics. Without honesty in these systems, selection should drive individuals to disbelieve conspecifics and assess them using other metrics.

Here, I model several types of assessment that are available to receivers. First, I vary whether receivers of information can simultaneously assess all potential displaying individuals, and second, I test whether the accumulation of effort into discrete items strengthens the accuracy of assessment by receivers. These options will help elucidate scenarios where we should more readily predict conspecific assessment of behavior.

*2. Entities, State Variables, and Scales*

Agents: In the model, the agents performing the behaviors are individuals moving on a continuous space landscape. In the model, there are two types of individuals; the first type is a signaler that will display in a certain spot to be observed by the second type. The first type can only display when it has found an item on the continuous landscape. The first type can display one at a time and not have this contribute to a larger effort that can be observed at any time in one build of the model. In the second build, an individual’s effort is logged over time and the cumulative effort can be observed by the second type. The second type can either observe all individuals simultaneously, or the second type has to observe individuals sequentially in time steps in certain builds of the model. Both types of individuals must feed, so part of their behavior is to forage if their energy reserves are too low.

Spatial units (display items): In the model the items to be retrieved follow simple, probabilistic growth dynamics and are represented by the grid cells. The probabilistic growth also allows me to vary the availability of items that individuals can use for display.

1. The initiation of display items starts with the number of “cells” multiplied by a small factor that creates these items. The items have an age that determines when they disappear if they reach that age and a hard carrying capacity. These items reproduce with a certain probability if the population numbers are below the carrying capacity.

Spatial units (food items): In the model the food items to be eaten follow simple, probabilistic growth dynamics and are represented by the grid cells. The probabilistic growth also allows me to vary the availability of items that individuals can use for display.

1. The initiation of display items starts with the number of “cells” multiplied by a small factor that creates these items. The items have an age that determines when they disappear if they reach that age and a hard carrying capacity. These items reproduce with a certain probability if the population numbers are below the carrying capacity.

Environment: To display and gain reputational benefits, the displaying individuals must return to the origin on the landscape where they have a “territory”. The audience area is where the receivers of the display return to if they do not need to forage.

Collectives (Breeds): There are two distinct types in all the builds of the model (see above for explanation).

*3. Process Overview and Scheduling*

Agent updating: Time is modeled in discrete steps where all of the agents go through a set of decision points before performing a behavior. All of the individuals are placed on the schedule from MASON, so that the order of agent action (including displayers, receivers, and both types of item) is randomized from step to step.

**Design Concepts**

*4.1 Basic Principles*

The basic principle underlying the model is the effectiveness of certain methods of display, and whether the display of multiple individuals can be assessed simultaneously. Specifically, in a total factorial set of model builds, displayers can either display item-by-item without being able to communicate past effort, or in contrast, having some sort of cumulative output that can be assessed. For the receivers of the display, these individuals can either assess all the displayers simultaneously or have to assess displayers individual by individual.

*4.2 Emergence*

The main output that should emerge from the model is a comparison of the list of estimates of each displayer provided by each of the receivers, compared to the actual output of displayers. Each of the lists provided by the receivers will be used to calculate a Spearman rank correlation coefficient, and the average of the correlation coefficients will be compared between the four main experiments.

*4.3 Adaptation*

There is no adaptation in the model.

*4.4 Objectives*

Individuals have to fulfill one main criteria before they can either display (displayers) or observer (receivers). Individuals must be above the energy threshold before they can perform any other behavior, and if they are below the energy threshold, will locate food in their immediate area and consume it, or move randomly in search of food.

*4.5 Learning*

Learning in this model is simple and takes place in the receivers who assess and log the effort of the displayers.

*4.6 Prediction*

There is no explicit prediction by individuals in this model.

*4.7 Sensing*

Individuals can sense where the display and food items are on the continuous landscape within a certain radius. The receivers can also sense the individuals in a certain radius to log if they are in display.

*4.8 Interaction*

Interactions in the model are indirect. Specifically, the individuals can acquire items on the landscape and thus prevent others from acquiring the item; however the individuals do not engage in direct competition.

*4.9 Stochasticity*

Several stochastic processes are used in the model. First, random processes dictate whether an item will appear in a certain grid cell. One of the main stochastic factors is whether receivers and displayers are in the center of the space at the same time. This allows the receivers to assess the output of displayers.

*4.10 Collectives*

There are no traditional collectives in the model.

*4.11 Observation*

The main observation in the model are the output lists from each of the receivers. The receivers all store a list that contains what they witness from the displayers. These lists are output in tabular format after a certain amount of time steps. The displayers also have a running log of how much they have performed and the displayers print out this data at the end of a model run. These two sets of values are compared using Spearman rank correlation coefficients to see if the receivers assessment of output is similar to the actual output of displayers.

These variables will be observed across a spectrum of values for one other variable. Specifically, these values will be tested against a varying spectrum of ecological scarcity of resources. I will vary the probability that items display on the landscape, thus increasing or decreasing the amount of time individuals must spend looking for food.

**Details**

*5.1 Initialization*

At t_0_ there are 20 individuals of each type. Between 160 and 400 display items are initialized onto the continuous space and between 160 and 400 display items are initialized onto the space. The ages of the display items and food items are randomly selected to produce a distribution of ages. The displayers are initialized onto their home “territory” (a spot on the continuous space that they will come back to display) and the receivers are initialized in random locations. The food reserves are randomly initiated for both types of individuals to produce a distribution of hunger.

*5.2 Input data*

No input data is used at this time.

*5.3 Submodels*

-Individual Submodels

The submodels for both types of individual will start similarly. First, individuals will enter their decision points by asking if their energy reserves are above their energy threshold. If their energy reserves are not higher than the threshold, the individuals will look for food in the vicinity, and if they can not find any food items, they will move in a random heading to search for food.

If the energy reserves of displayers are higher than the energy threshold, then the displayers will look for an item in the vicinity and return to the arena to display with the item.

If the energy reserves of the receivers are higher than the threshold, then they will return to the center of the space to observe the displayers in the vicinity, either simultaneously or sequentially.

All individuals remove 1 unit of energy no matter what behavior they perform.

-Grid Cell (grass and food items) Submodels

All items will increment their age and see if they are above the maximum age. If they are above the maximum age they will “die” by removing themselves from the landscape.
