# Supplementary material for: The Relative Effectiveness of Signaling Systems: Relying on External Items Reduces Signaling Accuracy while Leks Increase Accuracy
Source: PLoS One. 2014 Mar 13;9(3):e91725. doi: 10.1371/journal.pone.0091725 (PMC3953539; doi:10.1371/journal.pone.0091725)
Supplement: Model Validation S1 — The model validation describes the checks that were run on the model. The main components of the model were rigorously tested to confirm that the model was functioning as described. (DOCX) [file pone.0091725.s003.docx]

Model Validation Procedures For:

Discrete Items Enhance Reputation Assessment

Gavin M. Leighton^1^

^1^University of Miami, Department of Biology, 1301 Memorial Drive, Coral Gales, FL

Corresponding e-mail: [gleighton@bio.miami.edu](mailto:gleighton@bio.miami.edu)

*Outline*

The output assessment model is a spatially-explicit model where individuals have to collect food and display, or collect items to display, all while foraging for food items on a landscape. The crucial behavioral components of the model are the search for items (whether food or display items), the display of the items for displayers, the observation of display by receivers, and the memory of display by receivers. These components are verified using a series of model tests below.

The initialization of the model can be verified using a visual inspection of the built-in classes in MASON that allow for the visualization of the landscape. The initialization verification simply requires that appropriate numbers of displayers and receivers are created, and that the appropriate number of display and food items are created given a certain parameter set.

*Behavioral Validation*

**Displayers**: The displayers have to either look for food, or if they have enough energy reserves they can display on their home location. The foraging routine for both displayers and receivers is identical and will be addressed in the receivers’ validation (see below). For finding an item, individuals that were searching printed that they were about to search, and printed when they found an item. This was compared to the total number of display items to see if displayers were decrementing display items when they took them. In every case, a displayer that entered the search method for display items would look in its neighborhood for a display item, if the displayer found the display item, it would print that it found an item, print the number of display items, remove the item from the space, and then reprint the number of display items. Whenever a displayer needed a display item and found one, the display items were decremented by 1. The decrementing of 1 is important because the item being removed decrements the counter of items itself when it enters the removal method in it’s own set of behaviors, confirming that the item is being removed.

The next part is to confirm that displayers are moving back to their home location once they have acquired an item. To do this, three system printlines were used. First, the male printed his current location before moving, and then printed where he moved to followed by his set home location. In many cases, males could retrieve items from their home so that their locations didn’t change. The model behavior was validated though when displayers were not on their home territory, printed their location and home location, moved to their putative home, and reprinted their location after moving. In these cases, the new location always matched the given home location. This procedure also confirmed that males have different home territories.

The overall structure of the set of behaviors was confirmed by system printlines and having individuals print their own variable values. In each case, when their energy levels were below the threshold, they entered the foraging option. When an individual had sufficient energy reserves, they displayed if they had an item, and if not, they searched in the local vicinity for an item after moving randomly.

To confirm that displayers were incrementing their display variable if they had an item, each individual printed it’s cumulative variable during a time step and whether or not it had displayed. Indeed, in each case where an individual confirmed display, it’s cumulative display effort was appropriately incremented by 1.

**Receivers:** The receivers (and displayers) have to find food before they can observe. The foraging commands are identical between receivers and displayers, so their validation is listed here. The first step is to check the receivers’ energy reserves. The energy reserves were printed and compared to the threshold. If above the threshold, the individuals moved back to the display arena. When the reserves were below the threshold, the individual searched for food items in the vicinity. They placed these items into a list and randomly selected one item in the list. This was confirmed by printing the number and id of each item in the list. The individual printed the number of food items before consumption and after consumption. Similar to above, after consumption, the number of items is decremented by one after consumption, demonstrating that the item had been removed via the appropriate removal procedure in the foodItem class. Finally, the individual printed their energy reserves before and after consumption. In each case, the variable was updated by the appropriate energy increase.

When the energy reserves are high enough, the receivers should move to the center of the arena to observe males either sequentially or all at once. To confirm this, a system printline was used to confirm that receivers switched their location to the middle location (computed mathematically as ½ the width and ½ the height) after moving. This was also confirmed visually by watching the receivers move to the center.

A critical component for the receiver concerns assessing signalers and their output. This set of behaviors relies on similar code to the receivers and signalers finding items on the landscape. First each receiver has a list of values that will be used to assess the individuals signaling. This list has an index that is the same index (i.e. 0-19) given to the signalers. The code for assessing signalers depends on the type of model. In the first, receivers can assess the entire group of signalers in the arena and view their total output. This was validated by having the receivers print their list of values and comparing across the receivers. The receivers that are registering values should have the same exact values when observing individuals given that they can assess the full output. Indeed, the values matched between receivers. In the second, receivers can assess the entire group but only sees effort in real time. This was confirmed by having the individuals print lists and comparing lists. In each case, the same signalers were registered as having displayed.

To ensure some food or display item production, even if all the items are removed, there were two monitors that were created to seed new items. If there were 0 items on the landscape, these monitors created more items and randomly placed them on the landscape. This was confirmed visually and with printlines. The model was stepped by GML and when 0 items were left, the monitors created the expected value of items. This was confirmed using printlines by having the monitors print the number of items before and after the routine to check and create new items.

The receiver assessment of individuals varied with each model build, so that validation is required for each build. In the first build, the receivers could assess all the displayers simultaneously and could determine the cumulative effort of males.

In the second model build, the receivers could assess all of the displayers at once but could only incrementally update the value if they saw them in a single time step, instead of assessing cumulative effort. To confirm this, individual receivers printed out the individual they were observing and the value for that displayer. A series of printlines showed that receivers were recognizing individual displayers and incrementing their counts by one based on the presence of individuals.

In the third model build, individual receivers had to move from individual to individual to assess their output. To do this, the routine that placed displayers in a set of territories that formed a circle was used to have receivers move from territory to territory. In other words, if receivers had sufficient energy reserves, they would move from one territory to the next by incrementing the same functions that created the circular array of males. So that receivers could only assess one male at a time, the receivers could only assess displayers in a radius smaller than the distance to the next territory. This was also confirmed visually using the display, where individual receivers were observed moving in a circular motion from one territory to the next. In the third model build, receivers could assess the total output of individuals.

*Initialization Validation*

**Items**: When the number of food items is set to 400, and the number of display items is set to 600, a system print line is used to print the number of items (both types) initialized in the starting loops. In the output, 1-400 is output for the food initialization, and 1-600 is output for the display items. These sequences confirm that the number of items being created is numerically correct.

**Agents**: Similar to the items, a system printline was asked to print the total number of displayers and receivers after initialization. When the initial starting number for both receivers and displayers is set to 20, a series of 1-20 is printed twice, confirming that 20 displayers are created and 20 receivers are created. The displayers are also initialized in a small circle in the center of the discrete space. This allows each displayer to have a home location to display in where females can observe any males in the local space, i.e. any males that are on display.

**Neighborhood**: The distance with which the individuals can search is dictated by the “neighborhood” which is a predefined variable in the MASON toolkit. Specifically, the size of the neighborhood dictates how the discrete space is divided into space.
